# Intergeneric Relationships within the Early-Diverging Angiosperm Family Nymphaeaceae Based on Chloroplast Phylogenomics

**DOI:** 10.3390/ijms19123780

**Published:** 2018-11-28

**Authors:** Dingxuan He, Andrew W. Gichira, Zhizhong Li, John M. Nzei, Youhao Guo, Qingfeng Wang, Jinming Chen

**Affiliations:** 1 Key Laboratory of Aquatic Botany and Watershed Ecology, Wuhan Botanical Garden, Chinese Academy of Sciences, Wuhan 430074, China; hdxmusic@whu.edu.cn (D.H.); andrewgichira@gmail.com (A.W.G.); lizhizhong@wbgcas.cn (Z.L.); johnmulinge5@gmail.com (J.M.N.); qfwang@wbgcas.cn (Q.W.); 2 School of Biological and Pharmaceutical Engineering, Xinyang Agriculture and Forestry University, Xinyang 464000, China; 3 University of Chinese Academy of Sciences, Beijing 100049, China; 4 Sino-Africa Joint Research Center, Chinese Academy of Sciences, Wuhan 430074, China; 5 Laboratory of Plant Systematics and Evolutionary Biology, College of Life Sciences, Wuhan University, Wuhan 430072, China; yhguo@whu.edu.cn

**Keywords:** basal angiosperms, chloroplast, comparative genomics, Nymphaeales, Nymphaeaceae, phylogenomics, water lily

## Abstract

The order Nymphaeales, consisting of three families with a record of eight genera, has gained significant interest from botanists, probably due to its position as a basal angiosperm. The phylogenetic relationships within the order have been well studied; however, a few controversial nodes still remain in the Nymphaeaceae. The position of the *Nuphar* genus and the monophyly of the Nymphaeaceae family remain uncertain. This study adds to the increasing number of the completely sequenced plastid genomes of the Nymphaeales and applies a large chloroplast gene data set in reconstructing the intergeneric relationships within the Nymphaeaceae. Five complete chloroplast genomes were newly generated, including a first for the monotypic *Euryale* genus. Using a set of 66 protein-coding genes from the chloroplast genomes of 17 taxa, the phylogenetic position of *Nuphar* was determined and a monophyletic Nymphaeaceae family was obtained with convincing statistical support from both partitioned and unpartitioned data schemes. Although genomic comparative analyses revealed a high degree of synteny among the chloroplast genomes of the ancient angiosperms, key minor variations were evident, particularly in the contraction/expansion of the inverted-repeat regions and in RNA-editing events. Genome structure, and gene content and arrangement were highly conserved among the chloroplast genomes. The intergeneric relationships defined in this study are congruent with those inferred using morphological data.

## 1. Introduction

Considerable effort has been put into divulging the evolutionary origin of Angiosperms and, subsequently, significant progress has been made over the years [1,2,3,4,5,6,7,8]. The order Nymphaeales is currently considered as one of the early-diverging clades of Angiosperms, being the second group after Amborellales [2,4,9,10,11]. The circumscription of Nymphaeales varies from two families, Nymphaeales and Cabombaceae [12,13,14], to three families [15,16]. When included in the Nymphaeales, Hydatellaceae has been recognized as a sister to Nymphaeaceae [17].

Advances in molecular methodologies, especially the use of combined datasets, have led to significant strides towards attaining strongly resolved monophyletic clades within the three families of Nymphaeales. Cabombaceae is monophyletic, comprising of two genera, *Cabomba* and *Brasenia*, with strong support from both morphological and molecular datasets [17,18,19,20]. The twelve species of the Hydatellaceae family were initially placed into two genera, *Hydatella* and *Trithuria* [16], but were later combined into a single genus based on their reproductive characters and other morphological synapomorphies [21]. Uncertainties, however, exist concerning the monophyletic nature of Nymphaeaceae, more so in relation to the position of the *Nuphar* genus. This is despite the numerous studies aiming at reconstructing the phylogenetic relationships within the family.

Nymphaeaceae comprises of ca. 70 species that are classified under five genera [22], including *Nuphar* (~12), *Barclaya* (~4), *Euryale* (1), *Victoria* (2), and the largest and paraphyletic *Nymphaea* (~50 species). Phylogenetic analysis conducted on Nymphaeales, using fast evolving and noncoding chloroplast markers, weakly supported the monophyly of Nymphaeaceae and suggested several alternatives for the placement of *Nuphar* [19]. In another study, a combined approach of gene tree and species tree, based on a dataset of *matK* and ITS2, failed to give convincing support on the monophyly of the Nymphaeaceae family [17]. A more recent study [18] that analyzed 77 protein-encoded chloroplast genes, provided further compelling support to the monophyletic clades of Hydatellaceae and Cabombaceae. The study suggested alternative scenarios that placed *Nuphar* at varying positions, including as a sister clade to Cabombaceae and as a sister to a clade containing both Nymphaeaceae and Cabombaceae, which depicted Nymphaeaceae as paraphyletic.

The advancements made in DNA sequencing have accelerated the sequencing of chloroplast genomes [23] while the rapid progress in bioinformatics e.g., in [24] has facilitated downstream analyses of the generated sequences. Plastid genomes, compared to nuclear genomes, are relatively smaller and are abundantly present in a single cell, making it easier to extract, sequence, and fully annotate. Chloroplast genomes have low rates of nucleotide substitutions, they lack recombination, and mostly follow a non-Mendelian inheritance, making them more preferable for elucidating evolutionary relationships. In Nymphaeales, the mode of inheritance of the chloroplast DNA is exclusively uniparental. The prospective of chloroplast phylogenomics to resolve contentious phylogenetic relationships, at nearly all taxonomic levels, has been proven over the recent past, e.g., in providing strong support for the evolutionary clades of the basal Angiosperms [4,7], the early-diverging eudicots [8,25], and the early-diverging monocots [6]. Furthermore, through comparative phylogenomics, the availability of complete chloroplast genome sequences have significantly contributed to our understanding of genome evolutionary patterns driven by events such as gene transfers, duplications, and rearrangements [23,26].

Based on the most recent insights by Gruenstaeudl et al. [18], few species of the order Nymphaeales, only eight from four genera of Nymphaeaceae, have their complete chloroplast genomes sequenced. Increasing the number of taxa would significantly improve phylogenetic resolutions within Nymphaeaceae. In addition to an increased number of taxa, the choice of an outgroup is equally essential in resolving taxonomic relationships. In order to avoid long-branch artifacts and providing ambiguous inferences, the chosen outgroup should not be distantly related to the ingroup [27]. This study aimed at: (1) completely sequencing the plastid genomes of five Nymphaeaceae species; (2) characterizing the newly generated chloroplast genomes and examine codon usage, repeat sequences, and RNA-editing tendencies within Nymphaeaceae, (3) identifying the ideal rooting group and using it to, (4) elucidate the phylogenetic position of *Nuphar* and delimit intergeneric relationships within Nymphaeaceae family.

## 2. Results

### 2.1. Structure and Gene Content of the Chloroplast Genomes

**Figure 1:**
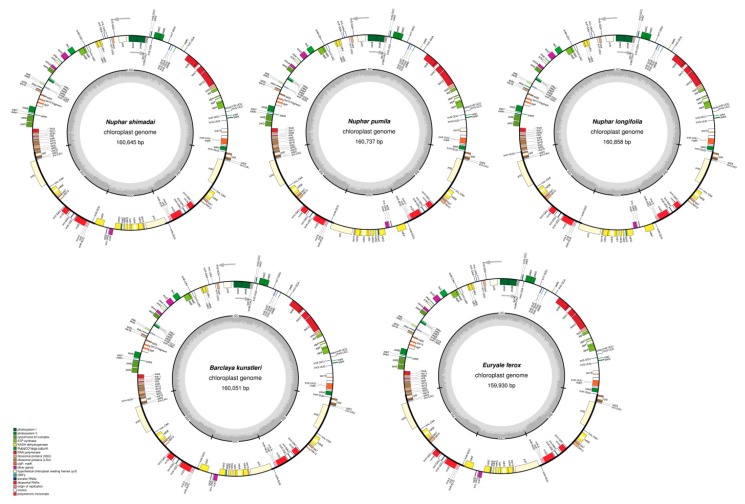
Circular gene maps of five chloroplast genomes of Nymphaeaceae. Grey arrows indicate the direction in which genes are transcribed. Color codes indicates the various gene functional groups, and the grey-shaded part in the inner circle shows the GC level of each chloroplast genome.

After discarding low-quality reads and sequence adaptors, 31,494,464–40,202,250 (99.82–99.94%) clean reads of 150 bp were generated for the newly sequenced species of Nymphaeaceae. The total length of the chloroplast genome sequence ranged from 159,930 bp in *E. ferox* to 160,858 bp in *N. longifolia* (Table 1, Figure 1). Identical to a majority of terrestrial plants, each of the five chloroplast genomes had two single copies of unequal length; a large single copy (LSC) and a small single copy (SSC), flanked by two equal inverted-repeat (IR) regions. Nucleotide composition with a GC content of 39.1% was nearly identical in all chloroplast genomes (Table 1).

A total of 113 unique genes were annotated in each of the newly reported chloroplast genomes, out of which 79 were protein-coding, 30 were transfer RNA, while four genes coded for the ribosomal RNAs (Table 2). In four of the species, 17 genes, including six PCGs, seven tRNAs, and four rRNAs, were wholly duplicated in the inverted-repeat regions. In *N. longifolia*, an extra gene, *trnH-GUG*, was located in the IRa region and was, therefore, entirely duplicated on IRb. In *N. pumila*, *N. shimadai*, and *B. kunstleri*, gene *trnH-GUG* was located at the IRb/LSC junction; thus, only a few base pairs of its 3′ end were duplicated in IRb. The coding region of 18 PCGs and tRNA genes was interrupted by either one or two introns (Table 2). The *rps12* gene has its 5′ exon in the LSC region, while two 3′ exons are duplicated in the IR region; thus, it was presumed to require trans-splicing during RNA processing.

### 2.2. Codon-Usage, RNA-Editing, and Repetitive-Sequence Analyses

**Figure 2:**
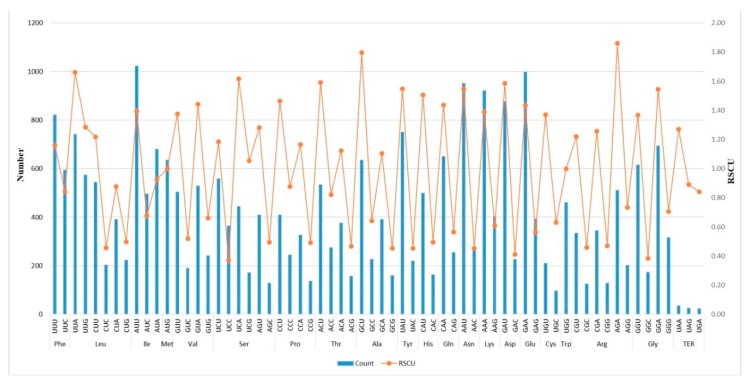
Details of codon preferences (bar) and relative synonymous codon usage values (line) of 12 chloroplast genomes of Nymphaeaceae.

Slight variations were observed in the usage of codons in all the analyzed species of Nymphaeaceae. Seventy-nine protein-coding genes in each of the chloroplast genomes, encoded between 26,126 and 26,378 codons (Table S2). In all the species, the amino acid leucine was encoded by the highest number of codons, ranging from 2669 in *N. pumila* to 2698 in *N. jamesoniana*. Cysteine was encoded by the least number of codons varying from 302 in the three newly reported species of *Nuphar* to 314 in *B. longifolia* and *N. advena.* As is normally the case with most angiosperms, only two codons, AUG for Methionine and UGG for Tryptophan, were used without any bias (relative synonymous codon usage (RSCU) = 1). The selection and usage of stop codons was biased in favor of TAA (RSCU > 1). The codons with A or T at their third positions were highly preferred to those with a C or G. In this regard, codon ATT for amino acid isoleucine (average 1023.58) had the highest count. Out of the 64 codons, 31 had RSCU values of more than 1; an indication that they were frequently used. The average number and RSCU for each codon was calculated for all 12 species (Figure 2; Table S2). The common initiation codon was ATG, although deviations were observed within some species, where GTG was noted in genes *rpoc1*, *cemA*, and *rps19*, while *psbL* and *ndhD* had ACG as the first codon.

**Figure 3:**
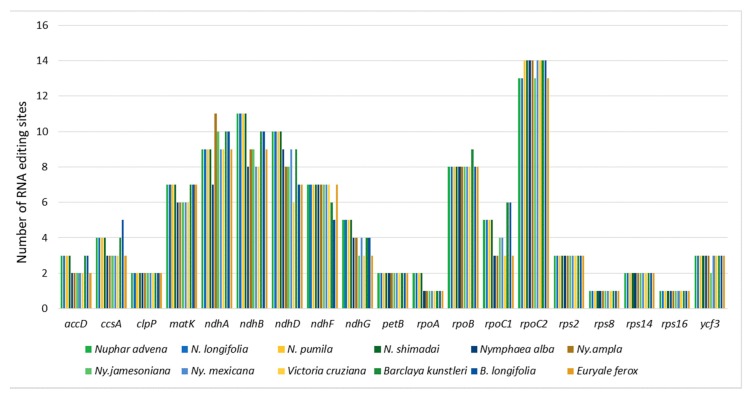
Number of RNA-editing sites in each of the transcripts of 19 common genes in all analyzed chloroplast genomes.

Potential RNA-editing sites were detected in between 24 and 28 protein-coding genes (Table 3; Table S3). All RNA-editing sites reported here were of the C to U type, the majority of which affected a single site, either the first or the second position of a given codon. However, in some genes, e.g., *ccsA* and *rpoC1*, only the third position was conserved in some codons. A total of 19 genes were commonly affected in each of the genomes. Out of these, *rpoC2*, *ndhA*, *ndhB*, and *ndhD* had the highest number of editing sites in each genome (Figure 3). In order to test for correlation between RNA-editing events and phylogenetic relationships, we used the details of the 19 common genes to create a binary data matrix that was then used to construct a UPGMA dendrogram in MEGA7 software (Figure S1).

A total of 438 short tandem repeats were mined in 12 species of Nymphaeaceae. The number of repeats in each chloroplast genome varied from 19 (*N. jamesoniana*) to 58 (*N. shimadai*). Interestingly, each species of *Nuphar* had a high number of repeats (>50), followed by the *Barclaya* species (>30). The majority of the microsatellites were A-T rich homopolymers, which was a common observation across all species except in *N. jamesoniana*, which had two strings of polyC (C10) and only one polyT (T13). Noncoding regions possessed more simple-sequence repeats (SSRs) compared to the coding regions. The repeat motif, length, and the location of the microsatellites are shown in Table S4. In addition, a total of 128 long tandem repeats were discovered in the 12 chloroplast genomes. Forty-nine repeats were found in the genome of *E. ferox*, which was the highest number of repeats in a single genome. Other genomes had between 12, in *N. pumila*, and one, in *B. longifolia*. Forward repeats exhibited a large percentage (80.5%), with the rest being the palindromic repeat sequences (Table S4).

### 2.3. Inverted Repeats and Genome Comparison

**Figure 4:**
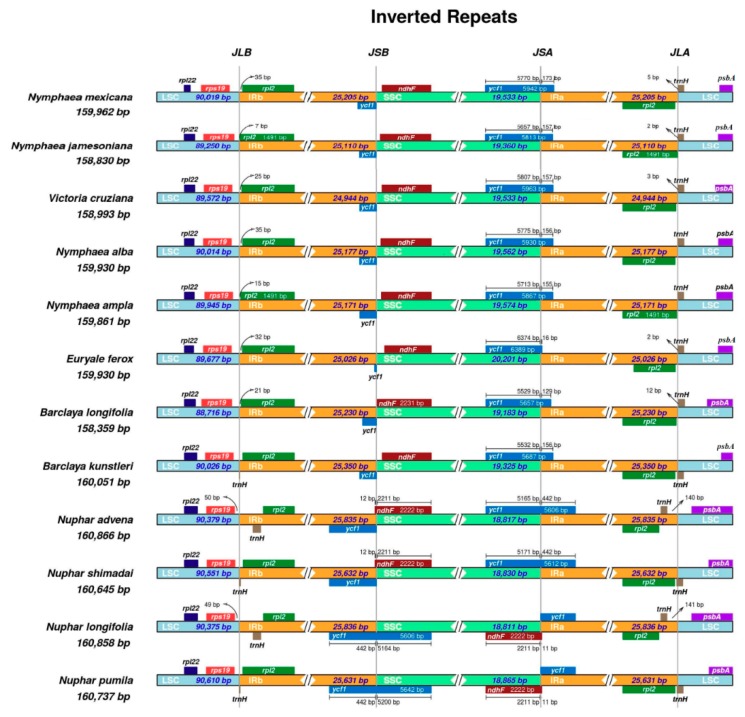
Comparison of the border positions of the large single copy, small single copy, and the inverted-repeat regions among chloroplast genomes of twelve species of Nymphaeaceae. Complete genes and portions of genes adjacent to the junctions are depicted by differently colored blocks.

**Figure 5:**
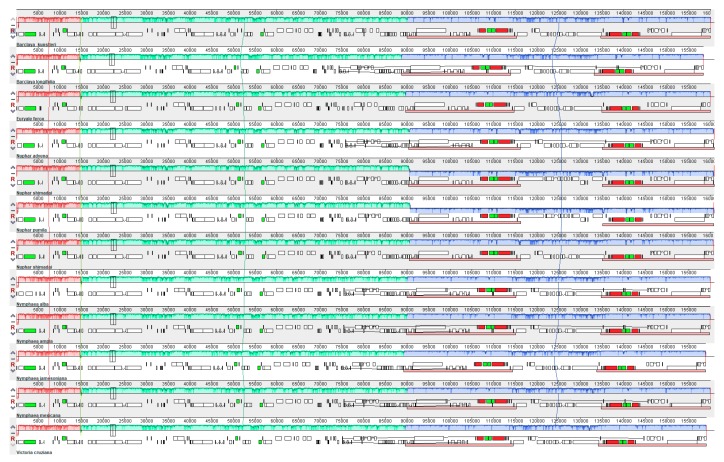
Mauve software alignment of the whole chloroplast genome of 12 species of Nymphaeaceae. Local collinear blocks representing identical gene clusters are depicted by the same color and are connected by lines.

Gene positioning at the IR/SSC junctions was stable, in that the JSA boundary expanded into the *ycf1* gene in all species at varying lengths, while the *ndhF* gene was squarely located in the IR, leaving a gap of varying length between JLB and the 3′ end of the gene. However, in all species of *Nuphar*, the JLB expanded into the *ndhF* gene, resulting in an overlap of 11 to 12 bp between the *ndhF* and the *ycf1* pseudogene and a relatively smaller SSC region compared to the other species. Significant variations were observed at the JLA and JLB junctions (Figure 4). Whole genome alignments, using Mauve, revealed well-conserved chloroplast genomes that lacked major inversions or rearrangements. Gene content and order were highly maintained and, thus, only three locally collinear blocks were identified among the species of Nymphaeaceae (Figure 5).

### 2.4. Phylogenetic Analyses

**Figure 6:**
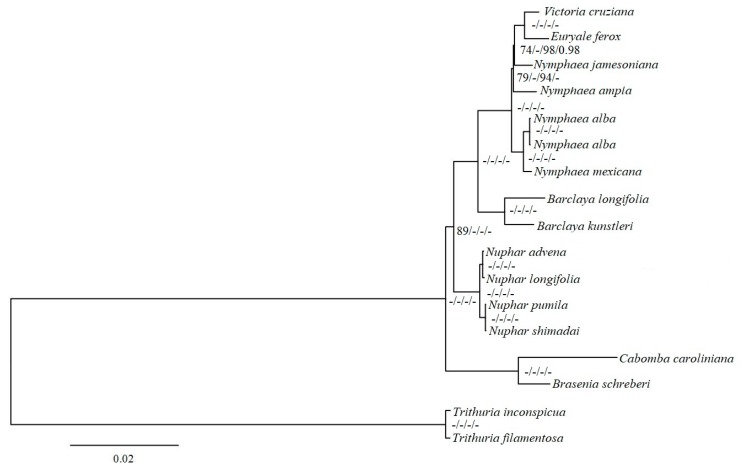
Phylogenetic relationships among the species of Nymphaeaceae, Cabombaceae, and Hydatellaceae (outgroup). The Maximum Likelihood (ML) and Bayesian Inference (BI) phylogenetic tree was based on 66 protein codon genes. The numbers indicate ML bootstrap support (100) and BI posterior probabilities (1.0) values. The - symbol indicates maximum support. The first two values and the last two are for unpartitioned data and partitioned data respectively.

The 66 protein-coding gene dataset produced highly congruent topologies based on the various Maximum Likelihood (ML) and Bayesian Inference (BI) strategies, using different partitioning approaches. Using *Amborella trichopoda* and under a partitioned data matrix, a weakly supported clade containing Cabombaceae and four genera of Nymphaeaceae was recovered; *Nuphar* was positioned (strongly supported) at the base and as a sister clade (Figure S2a). Under unpartitioned data, a clade containing *Nuphar* as a sister to Cabombaceae was strongly supported (Figure S2b). Using *Trithuria incospicua* and *T. filamentosa* (Hydatellaceae) as outgroup in the phylogenetic analyses, the monophyly of Nymphaeaceae and Cabombaceae were strongly supported (BS = 100, PP = 1.0) by both ML and BI phylogenetic analyses using unpartitioned and partitioned data matrix (Figure 6). The three newly generated species of *Nuphar* were fully supported as sisters to *N. advena* in a monophyletic clade. Similarly, *B. kunstleri* and *E. ferox* had full support at their respective nodes as sisters to *B. longifolia* and *V. cruziana*, respectively. The *Nymphaea* genus was strongly supported to be a paraphyletic clade in relation to *E. ferox* and *V. cruziana*.

## 3. Discussion

### 3.1. Chloroplast Genome Structure

Normally, chloroplast genomes of higher plants are highly conserved circular molecules with a size range of 120 to 160 kb, and they typically contain ~110–130 unique genes [28,29]. In this study, five recently sequenced complete chloroplast genome sequences on Nymphaeaceae were reported. These are added to the small but a steadily growing number of species whose chloroplast genomes have been reported in this family and in the Nymphaeales order. The overall structure, nucleotide composition, and gene content and arrangement among the reported taxa were nearly identical to each other and among those of early diverging angiosperms (Table 1 in this study, Table 1 in Gruenstaeudl et al. [18]). The genomes encoded an equal number of genes, 113 unique genes in total. The potential of the *ycf15* and *ycf68* genes to encode for protein in chloroplast genomes of basal angiosperms has previously been questioned [18,30,31]. Although sequences of the two hypothetical genes are well-preserved, partially or in whole in most of the species, studies suggest that these are not protein-coding genes and, therefore, were not annotated in the currently reported genomes. Likewise, the two open reading frames; *orf42* and *orf56*, which had been annotated in some genomes of Nymphaeaceae, were excluded in this study, based on the observation that their ability to code for proteins in Angiosperm is yet to be confirmed [18,32].

### 3.2. Codon-Usage, RNA-Editing, and Repetitive-Sequence Analyses

The genetic code in both eukaryotes and prokaryotes is degenerate and, with 61 codons encoding for only 20 amino acids, some amino acids are encoded by more than one codon [33]. Therefore, since codons are used with varying frequency, codon usage bias is generally inevitable. Codon usage is usually driven by mutational bias and natural selection [34], and the most-affected bases are usually at the third and sometimes at the second position of a codon, which was evident in the chloroplast genomes of Nymphaeaceae species. Normally, RSCU values greater than 1 indicate over-representation of a given codon, while values below 1 show less usage, and values of 1 indicate lack of bias in codon usage [35]. In each species, over 30 codons had RSCU >1, an indication that these were highly preferred and, as expected, all had A/T at their third position. Understanding codon-usage patterns may be effective in discerning the different evolutionary processes that affect chloroplast genomes. A well-preserved pattern of codon usage bias (CUB) was observed in all the studied species of Nymphaeaceae, which was nearly identical to those reported in other plant species [36,37].

RNA editing is typical in the chloroplast genome sequences of most land plants. The sequences are subject to regular modification at the transcript level through RNA editing and trans-splicing [38]. Thus, recognition of RNA-editing sites in transcripts is elemental for comprehending the coding patterns in chloroplast genomes. In addition, certain RNA-editing events cause divergence in the evolutionarily conserved amino acid sequences [39]. Here, we identified RNA editing sites in transcripts from each of the 12 complete chloroplast genomes of Nymphaeaceae. The number of editing sites varied slightly between 94 in *V. cruziana* and 108 in *B. kunstleri*. Although the majority of editing sites were in internal codons, the initiation codon ATG (amino acid methionine) was restored from ACG in the transcripts of genes *psbL*, *ndhB*, and *rpoC1*. There was no considerable difference in the number of genes affected by RNA editing, which varied from 24, in two species of *Nuphar*, to 28, in *B. kunstleri*. Nineteen genes were common in all the chloroplast genomes, and the majority of their editing sites were conserved. Comparative analyses have shown no correlation between RNA-editing events and phylogenetics in major groups of land plants [40]. However, in this study, further comparative analysis revealed certain patterns that are worth mentioning. For example, potential RNA-editing sites were predicted in the *atpB*, *atpI* and *rpl2* genes in all the genera except in *Nuphar*, while genes *psbF* and *petG* had no editing sites in the *Nymphaea*, *Victoria*, and *Euryale* clades. These patterns were also observed in the number of sites predicted in some gene transcripts, e.g., gene *accD* had three editing sites in *Nuphar*, and only two in all the other genera. The UPGMA dendrogram, constructed based on RNA-editing events, inferred a paraphyletic Nymphaeaceae supporting *Nuphar* as a sister to Cabombaceae. This implied that RNA-editing events are well-conserved genus/clade-specific evolutionary processes in the chloroplast genomes of Nymphaeaceae.

Repetitive sequences play various roles in genome organization, gene activities, and DNA recombination, replication, and repair [41]. Those located in the protein-coding regions may interfere with the normal functions of proteins [42]. The majority of the tandem repeats discovered in the chloroplast genomes of Nymphaeaceae were located in the noncoding segments. Short tandem repeats were plentifully distributed within genomes. Interestingly, species of *Nuphar*, with the largest genome sizes, exhibit the largest number of SSRs compared to the other genomes. In situations where SSRs are randomly distributed, more SSRs would be identified in larger chloroplast genomes compared to the smaller ones [31]. However, a positive correlation between genome size and the number of SSRs seems elusive, because *Nymphaea jamesoniana* had fewer SSR repeats than the species with the smallest genome size.

### 3.3. Comparative Analyses

Comparative chloroplast genomics provides insights into the evolutionary patterns of chloroplasts [23] and lays the foundation for functional genomic and phylogenomic studies [43]. The five genomes exhibit a quadripartite structure that is distinctive from the majority of land plants. Although chloroplast genomes are highly conserved, particularly among closely related species, minor variations are evident, and perhaps the most noticeable difference is the total genome size of the various species. Species of Nymphaeaceae have so far displayed a narrow range of size disparity, with *B. longifolia* (158,360 bp) [18] and *N. advena* (160,866 bp) [31] possessing the smallest and the largest genomes, respectively. Chloroplast genomes reported in this study differed slightly in size with a difference of about 1 kb between the smallest and the largest. The contraction/expansion of the inverted-repeat regions is listed among the main sources of size variations in chloroplast genomes. The IRs can greatly fluctuate in size and their positions differ even among species of the same genus [44]. The *Nuphar* genus harbors the largest chloroplast genomes among the Nymphaeaceae species and this is as a result of increased expansion of the IRs into the SC regions. In most chloroplast genomes of nonmonocot angiosperms, the *trnH-GUG* and *rps19* genes lie within the LSC region [44,45]. The JLA boundaries of *N. advena* and *N. longifolia*, the largest chloroplast genomes of Nymphaeaceae, are located upstream of *trnH-GUG* gene, which is, therefore, placed within the IRa region. The positioning of the IR/SC junction in these two species of *Nuphar* is congruent with the reports by Wang et al. [45], who, based on the results of *N. advena*, made a generalized observation for the Nymphaeaceae family. However, based on the results in this study, the positioning of the JLA and JLB junctions in Nymphaeaceae are rather more divergent. Other species whose genome sizes were over 160 kb, including *N. pumila*, *N. shimadai*, and *B. kunstleri*, had their IR/LSC expanded into the *trnH-GUG* gene, which, based on Wang et al. [45], belongs to the same category, (c), as some eudicots.

The mechanisms of expansion and contraction of the inverted-repeat regions have been shown to have evolutionary significance and could be used as sources of important molecular markers to elucidate relationships among various plant species [45,46]. The variations observed at the IR/LSC boundaries could be potential sources of phylogenetic markers ideal to study interfamilial relationships within Nymphaeaceae. The Mauve software combines the analysis of large-scale evolutionary events with traditional sequence alignments in order to identify conserved regions, rearrangements, and inversions in genomes [47]. The alignments revealed that the entire genome structure and gene arrangement are collinear and highly conserved within the Nymphaeaceae family. Only three locally collinear blocks were identified, which were interpreted to harbor three clusters of conserved homologous genes (Figure 5).

### 3.4. Phylogenetic Inference

Coding and noncoding regions of chloroplast genomes are subject to varying rates of molecular evolution, thus providing ample genetic variation for phylogenetic investigations at diverse taxonomic levels [48,49]. Phylogenetic analyses of one of the early diverging angiosperms, Nymphaeales, have been limited to the use of one or a few molecular markers obtained from plastid or nuclear genomes [13,17,19,50]. Consequently, they have provided important insight into the evolutionary relationship between major lineages of Nymphaeales. However, with the use of large-scale genome-wide datasets that have been made available by the rapidly increasing number of completely sequenced plastid genomes, well-resolved and strongly supported phylogenetic clades have been obtained [4,5,7]. The most recent phylogenetic analysis, utilizing multiple chloroplast protein-coding genes [18], strongly supported the monophyly of Cabombaceae and Hydatellaceae under different data partitions, but could not firmly support a monophyletic Nymphaeaceae family. In this study, more taxa of Nymphaeaceae were added to the eight used by Gruenstaeudl et al. [18]. Multigene phylogenetic analysis was conducted using 66 protein-coding genes obtained from 17 chloroplast genomes of Nymphaeales.

In spite of increased taxon sampling in *Nuphar*, its phylogenetic position remained vague in relation to the used outgroups. Using Amborellaceae to root the phylogenetic tree, both partitioned and unpartitioned data schemes placed *Nuphar* at two different positions, confirming earlier proposed hypotheses. Without data partitioning, *Nuphar* and Cabombaceae formed a weakly supported (44/0.5 BS/PP) clade that was sister to the rest of Nymphaeaceae, whereas, under partitioned data, *Nuphar* was positioned at the base, while Cabombaceae and the rest of Nymphaeaceae formed a weak (26/0.9 BS/PP) relationship (Figure S2). An outgroup provides evolutionary information, including more precise determination of pleisiomorphic traits of an ingroup [51]. Accordingly, an inappropriate choice of outgroup and limited taxon sampling may fail or give misleading phylogenetic resolutions [30,52,53].

The proximity of Hydatellaceae to the ingroup, containing Cabombaceae and Nymphaeaceae, makes it a fundamental root in defining character homology in *Nuphar* and the other genera. Consequently, our analyses provided strong statistical support for a monophyletic Nymphaeaceae, and resolutely confirmed the monophyly of Cabombaceae based on various data-partitioning schemes (Figure 6). In addition, the relationship between the five genera of Nymphaeaceae, *Nuphar*, *Barclaya*, *Nymphaea*, *Euryale*, and *Victoria*, was defined. *Nuphar* was placed at the base of the Nymphaeaceae family as a sister to *Barclaya*. These results are consistent with morphological circumscriptions of the family that places *Nuphar* at the basal position due to a lack of significant specialized features, synapomorphic for other Nymphaeaceae species [54,55]. Similarly, the clade consisting of *Barclaya*, *Nymphaea*, *Euryale*, and *Victoria* was strongly supported and congruent to Loehne et al. [19]. The relationship between the species of *Nuphar* corresponds to New World and Old World monophyletic subclades that were well-outlined and supported by both morphology and molecular datasets [55,56].

The *Barclaya* genus, endemic to Southeast Asia, was previously classified under a monotypic family, Barclayaceae, based on morphological traits, but was later moved to Nymphaeaceae based on cladistics and molecular evidence [57]. Within Nymphaeaceae, *Barclaya* was confirmed to be a close relative of *Nuphar* [54], a position that was strongly supported by genome-scale plastid data in this study (100%, ML and 1.0 PP, BI) under both partitioned and unpartitioned. *Nymphaea*, the largest and the most cosmopolitan genus within the family [58], has remained taxonomically challenging despite being accorded considerable attention. Although Borsch et al. [59] increased taxa sampling and improved molecular character sampling compared to the analysis done by Borsch et al. [59], certain nodes of *Nymphaea* subg. *Nymphaea* gained weak or lacked statistical support. In this study, a paraphyletic *Nymphaea* was strongly supported. However, certain internal nodes, such as the node linking *N. jamesoniana* and *N. ampla*, were moderately supported by ML analyses (BS = 79% and 94%, unpartitioned and partitioned data, respectively) despite being strongly supported by the BI (PP = 1.0). The currently used chloroplast DNA dataset has the potential to resolve these nodes but, to achieve this, extensive taxon sampling is needed.

The relationship of *Victoria* and the monotypic genus *Euryale* has long been accepted and supported by a combination of molecular and morphological data [54,60]. These two genera are associated by their aculeate character; their leaves are shieldlike, with petioles inserted at the center of the leaf blades, but they are easily distinguished based on the shape of the leaf margin and the presence or absence of staminodia and carpellary appendages [58]. Their relationship was strongly confirmed in this study, although their connection to *Nymphaea* was only moderately supported. Previous investigations firmly positioned *Victoria* within *Nymphaea* [18]. The addition of *Euryale* slightly reduced that support, although their position within *Nymphaea* was maintained. Strong conclusions concerning the phylogenetic and evolutionary relationships between the *Victoria–Euryale* clade and *Nymphaea* can only be made after more plastid genome data are made available.

## 4. Materials and Methods

### 4.1. Plant Material and Genome Sequencing

Fresh leaf samples of *Nuphar pumila*, *N. shimadai*, *N. longifolia*, and *Euryale ferox* were obtained from Wuhan Botanical Garden, Chinese Academy of Sciences, China, and voucher specimens were deposited in the Herbarium of the Wuhan Botanical Garden, Chinese Academy of Sciences (HIB). Leaf materials of *Barclaya kunstleri* were obtained from Bkt. Timah Natural Reserve, Singapore, and a voucher specimen was deposited in the Singapore Botanic Gardens Herbarium. About 5 g of fresh leaves per plant was collected and immediately dried with silica gel.

Total genomic DNA was isolated from 150 mg of silica-dried leaf tissues with DNeasy Plant Mini Kits (Tiangen, Beijing, China) following the manufacturer’s instructions. Approximately 5–10 μg of genomic DNA was used to construct paired-end sequencing libraries with insert sizes of between 250 and 350 bp for each species. These libraries were then sequenced using the Illumina Hiseq 2500 platform (Illumina Inc., San Diego, CA, USA) to generate at least 5 Gb of 300 bp paired-end read for all the species. The quality of the raw-sequence reads was checked using FastQC v0.11.2 (http://www.bioinformatics.babraham.ac.uk/projects/fastqc/), where ambiguous and low-quality reads were discarded.

### 4.2. Genome Assembly and Annotation

A reference-guided strategy was used to assemble the chloroplast genomes. In order to identify and retrieve the chloroplast sequences, the filtered reads were mapped to two reference chloroplast genomes, *Barclaya longifolia* (KY_284156) [18] and *Nuphar advena* (NC_008788) [31], using Bowtie2 2.2.9 [61] with parameters D 15-R 2-N 1-L 22-i S,1,1.15. The extracted reads were assembled de novo using Velvet 1.2.10 [62] with the following settings: velveth K = 79~105 and velvetg cov_cutoff = 100, resulting into 3–5 contigs. All contigs of each species were mapped to reference using GENEIOUS R8 (Biomatters Ltd., Auckland, New Zealand). The overlapping ends, 50–80 bp, were trimmed, while the gaps were filled by PCR amplification and Sanger sequencing using specifically designed primers. The positions of the single copies and the inverted-repeat regions were confirmed through self-blasting using Basic Local Alignment Search Tool (BLAST+). To verify the generated contigs, the reads were remapped to the complete chloroplast genomes using Bowtie2 2.2.9 with default parameters [61].

Each of the assembled chloroplast genomes was annotated using GeSeq [63] and Dual Organellar GenoMe Annotator (DOGMA) [64] using *B. longifolia* and *N. advena* as references. The annotations were manually corrected, wherever necessary, and verified using GENEIOUS R8 (Biomatters Ltd., Auckland, New Zealand) by realigning with the references. Finally, graphical circular gene maps for each of the species were constructed using OGDraw v1.2 [65]. The fully annotated chloroplast genomes were submitted to GenBank (accession numbers are shown in Table 1).

### 4.3. Chloroplast Genome Comparisons

In order to discover any significant interspecific and intergeneric variations among the newly generated chloroplast genome sequences of Nymphaeaceae, comparison analyses were carried out, focusing on various characters of the genomes, including sizes and gene content. The variations observed in chloroplast genome sizes are largely attributed to the contraction or expansion of the inverted regions. The four IR/SC borders of each of the chloroplast genomes of 12 species of Nymphaeaceae (five newly generated chloroplast genomes in this study and seven previous published chloroplast genomes: GenBank accession NC_008788, KU234277, KU189255, NC_024542, NC_031826, KY284156, and KY001813) and their adjacent genes were compared. Further, we used Mauve genome-alignment software [47] to conduct multiple genome-alignment analysis aiming at detecting any rearrangements or inversions within the chloroplast genomes of the 12 species of Nymphaeaceae.

### 4.4. Codon-Usage, RNA-Editing and Repetitive Sequences Analyses

The annotations errors in *Nymphaea jamesoniana*, as highlighted by Gruenstaeudl et al. [18], were corrected, and genes *ycf15* and *ycf68* and the two open reading frames (*orf42* and *orf56*) were excluded from these analyses. *Nymphaea alba* had two GenBank accessions, KU234277 and NC006050; we randomly picked KU234277 for these analyses. The frequency of synonymous codon usage, also referred to as CUB, was determined for all exons of 79 protein-coding genes in 12 species of Nymphaeaceae using MEGA Version 7 software [66]. The values of RSCU [67] were compared. Potential RNA-editing positions in the protein-coding genes of each chloroplast genome were predicted using Predictive RNA Editor for Plants (PREP) [68]. PREP uses 35 protein genes as reference to predict C-to-U editing events. The cut-off value was set at 0.8. MIcroSAtellite identification tool (MISA) [69] was used to search for SSR). The minimal repeat numbers were set at 10 for mono-, 5 for di-, 4 for tri-, and 3 for tetra-, penta-, and hexa-nucleotide repeat motifs. We used REPuter [70] to establish the size and location of direct, inverted, compliment, and reverse-repeat units in each of the chloroplast genomes of Nymphaeaceae. The lower limit of repeat size was set at 30 bp, with a repeat identity of 90% and a hamming distance of 3.

### 4.5. Phylogenetic Analyses

All currently available complete chloroplast genome sequences of Nymphaeaceae, which represented four genera, were retrieved from GenBank (Table S1). Five new genomes were reported in this study, including a first for the *Euryale* genus. Chloroplast genome sequences of two genera of Cabombaceae, two species of Schisandraceae, two representatives of the monotypic Hydatellaceae family, and the monotypic genus *Amborella* were also obtained (Table S1). Several phylogenetic analyses using *Amborella* or Hydatellaceae as outgroup were conducted to determine the effects of outgroup selection on the taxonomic relationships within Nymphaeaceae and Cabombaceae.

Sixty-six protein-coding genes, common in all genome sequences, were extracted and aligned using the Muscle program [71]. The aligned sequences were concatenated, and topologies were constructed using ML and Bayesian Inference conducted in RAxML v.8.2.9 [72] and MrBayes v.3.2.5 [73]. The best-fitting nucleotide substitution models based on the Akaike information criterion were realized using jModeltest v.2.1.7 [74]. ML analyses were conducted using a GTR + G + I substitution model with 1000 bootstrap replicates. A heuristic search of 10 independent replicates was carried out for the ML analyses. BI analysis was done using the GTR + G model and, based on the Markov chain Monte Carlo (MCMC) algorithm, one million generations with four independent heated chains with sampling after every 1000 generations. Convergence was attained and operation stopped when the average standard deviation of split frequencies remained below 0.01. The initial 25% of all sampled trees were discarded as burn-in, while the remaining 75% were used to construct a majority-rule consensus tree with posterior probabilities.

We conducted further phylogenetic analyses based on two different data partitions under ML and BI strategies. In the first phylogenetic analysis, we used jModeltest. v.2.1.7 [74] to infer the best-fitting substitution model for each of the 66 used genes. In this approach, each of the 66 genes was analyzed as a single partition. In the second analysis, the greedy-search algorithm executed in PartitionFinder2 [75] was used to determine the best model among the GTR, GTR + G, and GTR + I + G models based on the corrected selection criterion, the Aikaike Information Criterion (AICc).

## 5. Conclusions

Five newly sequenced complete chloroplast genomes of Nymphaeaceae, including the first in the *Euryale* genus, were reported. Comparative genomics revealed highly conserved patterns in relation to genome structure, nucleotide composition, and relative synonymous codon usage. However, minor variations were evident, particularly in the contraction/expansion of the inverted-repeat regions and in RNA-editing events, the majority of which appeared to be genus-specific, implying that each genus could have been subjected to unique evolutionary events. This study affirms the potential of chloroplast phylogenomics to solve taxonomic relationships within genera of Nymphaeaceae. By increasing taxa number and analyzing the validities of outgroups, a monophyletic Nymphaeaceae was attained, and the phylogenetic position of *Nuphar* was ascertained with strong statistical support. Nonetheless, there is need for further investigations to corroborate these findings.

## Figures and Tables

**Table 1:** Characteristics of chloroplast genomes of five species of Nymphaeaceae.

Name of Organism	*Barclaya kunstleri*	*Euryale ferox* Salisb.	*Nuphar longifolia* (Michx.) Sm.	*Nuphar pumila* (Timm) DC.	*Nuphar shimadai* Hayata
GenBank accession number	KY392762	KY392765	MH050795	MH050796	MH050797
Genome size (bp)	160,051	159,930	160,858	160,737	160,645
Large single copy (LSC) length (bp)	90,026	89,677	90,375	90,610	90,551
Small single copy (SSC) length (bp)	19,325	20,201	18,811	18,865	18,830
Inverted repeat (IR) length (bp)	25,350	25,026	25,836	25,631	25,632
Number of genes	113	113	113	113	113
Number of protein-coding genes (duplicated in IR)	79 (6)	79 (6)	79 (6)	79 (6)	79 (6)
Number of tRNA genes (duplicated in IR)	30 (7)	30 (7)	30 (8)	30 (7)	30 (7)
Number of rRNA genes (duplicated in IR)	4 (4)	4 (4)	4 (4)	4 (4)	4 (4)
Number of genes with one intron (two introns)	15 (3)	15 (3)	15 (3)	15 (3)	15 (3)
Proportion of coding to noncoding regions	0.68	0.68	0.69	0.68	0.69
Average gene density (genes/kb)	0.82	0.82	0.83	0.82	0.82
GC content (%)	39.1	39.1	39.1	39.1	39.1

**Table 2:** List of genes encoded in each of the five chloroplast genomes of Nymphaeaceae.

Category	Gene Type	Gene						
Self-replication	Ribosomal RNA	*rrn16*	*rrn23*	*rrn4.5*	*rrn5*			
	Transfer RNA	*trnA-UGC* *	*trnfM-CAU*	*trnI-GAU* *	*trnM-CAU*	*trnR-ACG*	*trnS-UGA*	
		*trnC-GCA*	*trnG-GCC*	*trnK-UUU* *	*trnN-GUU*	*trnW-CCA*	*trnT-GGU*	
		*trnD-GUC*	*trnG-UCC* *	*trnL-CAA*	*trnY-GUA*	*trnR-UCU*	*trnT-UGU*	
		*trnE-UUC*	*trnH-GUG*	*trnL-UAA* *	*trnP-UGG*	*trnS-GCU*	*trnV-GAC*	
		*trnF-GAA*	*trnI-CAU*	*trnL-UAG*	*trnQ-UUG*	*trnS-GGA*	*trnV-UAC* *	
	Small ribosomal units	*rps11*	*rps12*	*rps14*	*rps15*	*rps16* *	*rps18*	
		*rps19*	*rps2*	*rps3*	*rps4*	*rps7*	*rps8*	
	Large ribosomal units	*rpl14*	*rpl16*	*rpl2* *	*rpl20*	*rpl22*	*rpl23*	*rpl32*
		*rpl33*	*rpl36*					
	RNA polymerase subunits	*rpoA*	*rpoB*	*rpoC1* *	*rpoC2*			
	translation initiation factor	*infA*						
Photosynthesis genes	NADH dehydrogenase	*ndhA* *	*NdhB* *	*ndhC*	*ndhD*	*ndhE*	*ndhF*	
		*ndhG*	*ndhH*	*ndhI*	*ndhJ*	*ndhK*		
	photosystem I	*psaA*	*psaB*	*psaC*	*psaI*	*psaJ*	*ycf3* **	*ycf4*
	photosystem II	*psbA*	*psbB*	*psbC*	*psbD*	*psbE*	*psbF*	*psbH*
		*psbI*	*psbJ*	*psbK*	*psbL*	*psbM*	*psbN*	*psbT*
		*psbZ*						
	cytochrome b/f complex	*petA*	*petB*	*petD*	*petG*	*petL*	*petN*	
	ATP synthase	*atpA*	*atpB*	*atpE*	*atpF* *	*atpH*	*atpI*	
	Large subunit of rubisco	*rbcL*						
Other genes	Maturase	*matK*						
	Protease	*clpP* **						
	Acetyl-CoA-carboxylase sub-unit	*accD*						
	Envelope membrane protein	*cemA*						
	Component of TIC complex	*ycf1*						
	c-type cytochrome synthesis	*ccsA*						
Unknown	hypothetical genes reading frames	*ycf2*						

Notes: the * and ** symbols indicate genes with one and two intron(s) respectively.

**Table 3:** List of protein-coding genes affected by RNA editing in each of the 12 chloroplast genomes of Nymphaeaceae.

*Nuphar advena*	*Nuphar longifolia*	*Nuphar pumila*	*Nuphar shimadai*	*Nymphaea alba*	*Nymphaea ampla*	*Nymphaea jamesoniana*	*Nymphaea mexicana*	*Victoria cruziana*	*Euryale ferox*	*Barclaya kunstleri*	*Barclaya longifolia*
*accD* ^3^	*accD* ^3^	*accD* ^3^	*accD* ^3^	*accD* ^2^	*accD* ^2^	*accD* ^2^	*accD* ^2^	*accD* ^2^	*accD* ^2^	*accD* ^3^	*accD* ^3^
*atpA*	*atpA*	*atpA*	*atpA*	*AtpA*		*atpA*	*atpA*	*atpA*	*atpA*	*atpA*	*atpA*
				*atpB*	*atpB*	*atpB*	*atpB*	*atpB*	*atpB*	*atpB*	*atpB*
						*atpF*					
				*atpI*	*atpI*	*atpI*	*atpI*	*atpI*	*atpI*	*atpI*	*atpI*
*ccsA* ^4^	*ccsA* ^4^	*ccsA* ^4^	*ccsA* ^4^	*ccsA* ^3^	*ccsA* ^3^	*ccsA* ^3^	*ccsA* ^3^	*ccsA* ^3^	*ccsA* ^3^	*ccsA* ^4^	*ccsA* ^5^
*clpP* ^2^	*clpP* ^2^	*clpP* ^2^	*clpP* ^2^	*clpP* ^2^	*clpP* ^2^	*clpP* ^2^	*clpP* ^2^	*clpP* ^2^	*clpP* ^2^	*clpP* ^2^	*clpP* ^2^
*matK* ^7^	*matK* ^7^	*matK* ^7^	*matK* ^7^	*matK* ^6^	*matK* ^6^	*matK* ^6^	*matK* ^6^	*matK* ^6^	*matK* ^7^	*matK* ^7^	*matK* ^7^
*ndhA* ^9^	*ndhA* ^9^	*ndhA* ^9^	*ndhA* ^9^	*ndhA* ^7^	*ndhA* ^11^	*ndhA* ^10^	*ndhA* ^9^	*ndhA* ^9^	*ndhA* ^9^	*ndhA* ^10^	*ndhA* ^10^
*ndhB* ^11^	*ndhB* ^11^	*ndhB* ^11^	*ndhB* ^11^	*ndhB* ^8^	*ndhB* ^9^	*ndhB* ^9^	*ndhB* ^8^	*ndhB* ^8^	*ndhB* ^9^	*ndhB* ^10^	*ndhB* ^10^
*ndhD* ^10^	*ndhD* ^10^	*ndhD* ^10^	*ndhD* ^10^	*ndhD* ^9^	*ndhD* ^8^	*ndhD* ^8^	*ndhD* ^9^	*ndhD* ^6^	*ndhD* ^7^	*ndhD9*	*ndhD* ^7^
*ndhF* ^7^	*ndhF* ^7^	*ndhF* ^7^	*ndhF* ^7^	*ndhF* ^7^	*ndhF* ^7^	*ndhF* ^7^	*ndhF* ^7^	*ndhF* ^7^	*ndhF* ^7^	*ndhF* ^6^	*ndhF* ^5^
*ndhG* ^5^	*ndhG* ^5^	*ndhG* ^5^	*ndhG* ^5^	*ndhG* ^4^	*ndhG* ^4^	*ndhG* ^3^	*ndhG* ^4^	*ndhG* ^3^	*ndhG* ^3^	*ndhG* ^4^	*ndhG* ^4^
*petB* ^2^	*petB* ^2^	*petB* ^2^	*petB* ^2^	*petB* ^2^	*petB* ^2^	*petB* ^2^	*petB* ^2^	*petB* ^2^	*petB* ^2^	*petB* ^2^	*petB* ^2^
		*petD*									
*petG*	*petG*	*petG*	*petG*							*petG*	*petG*
*psbE*	*psbE*	*psbE*	*psbE*		*psbE*	*psbE*	*psbE*	*psbE*	*psbE*	*psbE*	*psbE*
*psbF*	*psbF*	*psbF*	*psbF*							*psbF*	*psbF*
*psbL*	*psbL*			*psbL*	*psbL*	*psbL*	*psbL*	*psbL*	*psbL*	*psbL*	*psbL*
				*rpl2*	*rpl2*	*rpl2*	*rpl2*	*rpl2*	*rpl2*	*rpl2*	*rpl2*
*rpl20*	*rpl20*	*rpl20*	*rpl20*	*rpl20*	*rpl20*	*rpl20*	*rpl20*	*rpl20*	*rpl20*	*rpl20*	
*rpoA* ^2^	*rpoA* ^2^	*rpoA* ^2^	*rpoA* ^2^	*rpoA* ^1^	*rpoA* ^1^	*rpoA* ^1^	*rpoA* ^1^	*rpoA* ^1^	*rpoA* ^1^	*rpoA* ^1^	*rpoA* ^1^
*rpoB* ^8^	*rpoB* ^8^	*rpoB* ^8^	*rpoB* ^8^	*rpoB* ^8^	*rpoB* ^8^	*rpoB* ^8^	*rpoB* ^8^	*rpoB* ^8^	*rpoB* ^8^	*rpoB* ^8^	*rpoB* ^8^
*rpoC1* ^5^	*rpoC1* ^5^	*rpoC1* ^5^	*rpoC1* ^5^	*rpoC1* ^3^	*rpoC1* ^3^	*rpoC1* ^4^	*rpoC1* ^4^	*rpoC1* ^3^	*rpoC1* ^3^	*rpoC1* ^6^	*rpoC1* ^6^
*rpoC2* ^13^	*rpoC2* ^13^	*rpoC2* ^14^	*rpoC2* ^14^	*rpoC2* ^14^	*rpoC2* ^14^	*rpoC2* ^13^	*rpoC2* ^14^	*rpoC2* ^14^	*rpoC2* ^13^	*rpoC2* ^14^	*rpoC2* ^14^
*rps2* ^3^	*rps2* ^3^	*rps2* ^3^	*rps2* ^3^	*rps2* ^3^	*rps2* ^3^	*rps2* ^3^	*rps2* ^3^	*rps2* ^3^	*rps2* ^3^	*rps2* ^3^	*rps2* ^3^
*rps8* ^1^	*rps8* ^1^	*rps8* ^1^	*rps8* ^1^	*rps8* ^1^	*rps8* ^1^	*rps8* ^1^	*rps8* ^1^	*rps8* ^1^	*rps8* ^1^	*rps8* ^1^	*rps8* ^1^
*rps14* ^2^	*rps14* ^2^	*rps14* ^2^	*rps14* ^2^	*rps14* ^2^	*rps14* ^2^	*rps14* ^2^	*rps14* ^2^	*rps14* ^2^	*rps14* ^2^	*rps14* ^2^	*rps14* ^2^
*rps16* ^1^	*rps16* ^1^	*rps16* ^1^	*rps16* ^1^	*rps16* ^1^	*rps16* ^1^	*rps16* ^1^	*rps16* ^1^	*rps16* ^1^	*rps16* ^1^	*rps16* ^1^	*rps16* ^1^
*ycf3* ^3^	*ycf3* ^3^	*ycf3* ^3^	*ycf3* ^3^	*ycf3* ^3^	*ycf3* ^3^	*ycf3* ^2^	*ycf3* ^3^	*ycf3* ^3^	*ycf3* ^3^	*ycf3* ^3^	*ycf3* ^3^

Note: the superscript number indicates the number of edited sites in each of the 19 protein-coding genes common in all the genomes.

## Supplementary Materials

The following are available online at http://www.mdpi.com/1422-0067/19/12/3780/s1. Figure S1. UPGMA dendrogram of the species of Nymphaeaceae, Cabombaceae, and Hydatellaceae constructed based on the events of RNA editing in the transcripts of 19 common protein-coding genes. Figure S2. Phylogenetic relationships of the species of Nymphaeales based on the (a) partitioned and (b) unpartitioned data scheme of 66 protein-coding genes. *Amborella trichopoda* was used as an outgroup. The numbers indicate ML bootstrap support (100) and BI posterior probabilities (1.0) values. The * symbol indicates maximum support. Table S1. Details of taxa used in phylogenetic analyses. Table S2. Details of codon usage and relative synonymous codon usage values of chloroplast genomes of 12 species of Nymphaeaceae. Table S3. Details of RNA-editing events, including genes whose transcripts were affected in each genome and the number of editing sites in each gene transcript. Table S4. Details of short and long repetitive sequences discovered in each of the chloroplast genomes of 12 species of Nymphaeaceae.

## Abbreviations

IR	Inverted repeat.
SSC	Small single copy.
LSC	Large single copy.
SSR	Simple sequence repeat.
PCGS	Protein-coding genes.
RNA	Ribonucleic acid
RSCU	Relative synonymous codon usage
CUB	Codon usage bias
UPGMA	Unweighted pair group method with arithmetic mean
ML	Maximum likelihoodBI Bayesian inference
PP	Posterior probability
