# Supplementary material for: Intergeneric Relationships within the Early-Diverging Angiosperm Family Nymphaeaceae Based on Chloroplast Phylogenomics
Source: Int J Mol Sci. 2018 Nov 28;19(12):3780. doi: 10.3390/ijms19123780 (PMC6320877; doi:10.3390/ijms19123780)
Supplement: Supplementary file 1 [file ijms-19-03780-s001.zip › Supplemental files/Table S1.docx]

| **Species** | **Family** | **GenBank Accession number** |
| --- | --- | --- |
| *Nuphar advena* W.T. Aiton | Nymphaeaceae | NC_008788 |
| *Nymphaea alba* L. | Nymphaeaceae | KU234277 |
| *Nymphaea alba* L. | Nymphaeaceae | NC_006050 |
| *Nymphaea ampla* DC | Nymphaeaceae | KU189255 |
| *Nymphaea mexicana* Zucc. | Nymphaeaceae | NC_024542 |
| *Nymphaea jamesoniana* Planch. | Nymphaeaceae | NC_031826 |
| *Barclaya longifolia* Wall. | Nymphaeaceae | KY284156 |
| *Victoria cruziana* Orb. | Nymphaeaceae | KY001813 |
| *Brasenia schreberi* J.F.Gmel. | Cabombaceae | NC_031343 |
| *Cabomba caroliniana* A.Grey | Cabombaceae | KT705317 |
| *Trithuria inconspicua* Cheeseman | Hydatellaceae | NC_020372 |
| *Trithuria filamentosa* Rodway | Hydatellaceae | KF696682 |
| *Amborella trichopoda* Baill. | Amborellaceae | NC_005086 |
| *Schisandra chinensis* (Turcz.) K. Koch | Schisandraceae | KU362793 |
| *Illicium oligandrum* Merr. and Chun | Schisandraceae | NC_009600 |

**Table S1.** Details of species of the order Nymphaeales used in phylogenomics analysis
