# Supplementary figures and images for: Intergeneric Relationships within the Early-Diverging Angiosperm Family Nymphaeaceae Based on Chloroplast Phylogenomics
Source: Int J Mol Sci. 2018 Nov 28;19(12):3780. doi: 10.3390/ijms19123780 (PMC6320877; doi:10.3390/ijms19123780)

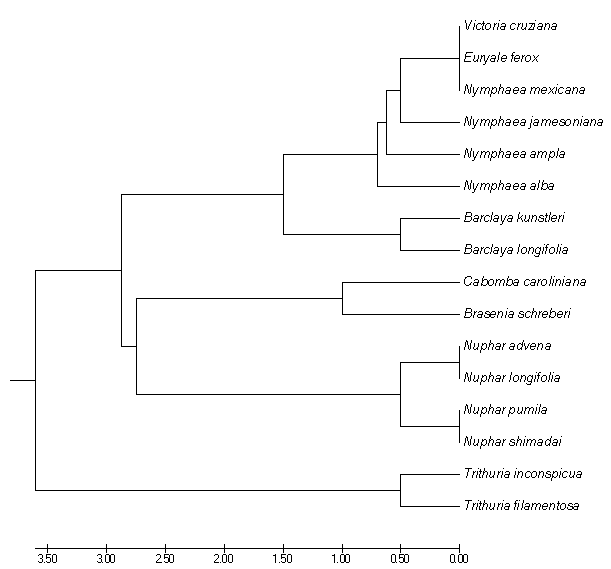

Supplement: Supplementary file 1 [file ijms-19-03780-s001.zip › Supplemental files/Figure_S1.jpg]

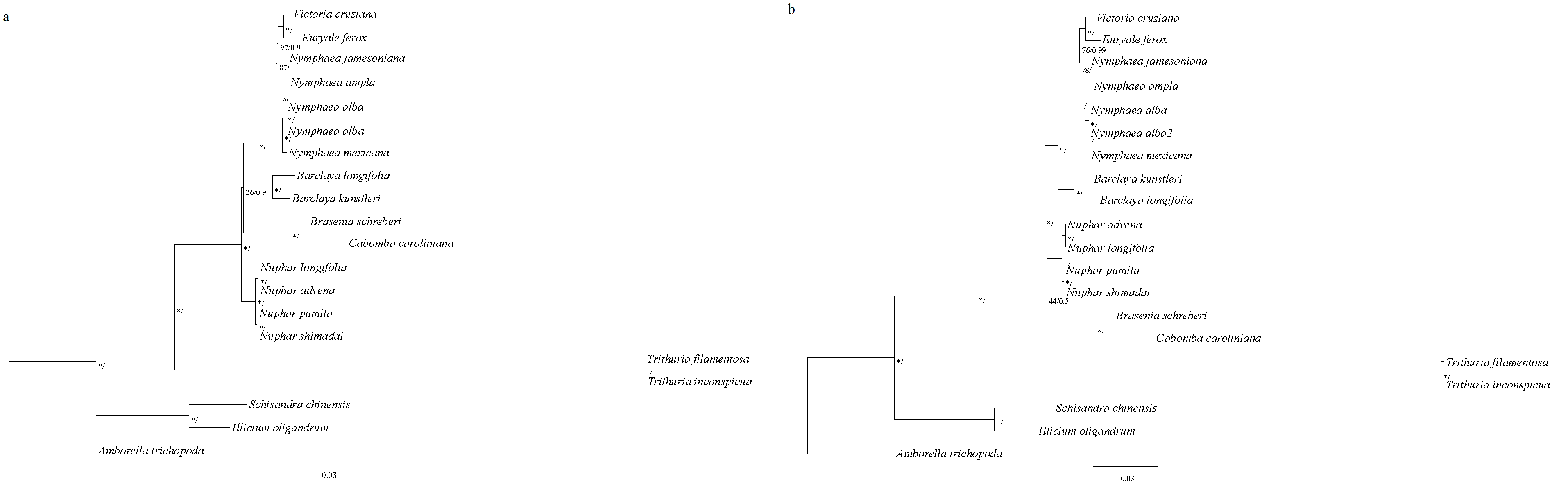

Supplement: Supplementary file 1 [file ijms-19-03780-s001.zip › Supplemental files/Figure_S2.jpg]
